# P43 Unmasking posterior reversible encephalopathy syndrome: a hidden threat in juvenile lupus

**DOI:** 10.1093/rap/rkae117.074

**Published:** 2024-11-05

**Authors:** Ola Saad, Nadia Rafiq

**Affiliations:** Evelina London Children Hospital, London, United Kingdom; Evelina London Children Hospital, London, United Kingdom

## Abstract

**Introduction:**

Posterior reversible encephalopathy syndrome (PRES) is a neurological condition identifiable through clinical presentation and magnetic resonance imaging (MRI). While PRES has been rarely documented in children as a complication of juvenile lupus, it remains a potentially reversible condition. However, it can be complicated by vasculopathy, infarction, or haemorrhage. The pathogenesis of PRES is still unclear. In juvenile lupus, the risk factors for PRES are multifaceted and interact with each other, including vasculitis, lupus nephritis, chronic kidney disease (CKD) leading to renal-origin hypertension, and iatrogenic hypertension caused by several hypertension-inducing drugs commonly used as first-line treatments for lupus.

**Case description:**

A 14-year-old girl diagnosed with lupus presented with persistent fatigue, lower limb oedema, and abdominal symptoms (vomiting and diarrhoea). She experienced two episodes of PRES, three months apart. Active SLE was confirmed through clinical and laboratory findings, including positive autoimmune and serologic results for ENA antibody screen, high levels of anti-double-stranded DNA (>400), and low complements C3 (0.15 g/L) and C4 (<0.03 g/L). Urine analysis revealed 2+ RBCs, 3+ proteins, and an estimated glomerular filtration rate (eGFR) of ∼21 ml/min/1.73 m² with a plasma creatinine of 226 micromol/L. Renal biopsy identified lupus nephritis class IV and chronic kidney disease (CKD) changes, leading to a diagnosis of CKD stage 4.

Initial treatment included a course of pulse methylprednisolone (1 g daily for 3 days) alongside rituximab and MMF for remission induction followed by high-dose oral prednisone (2 mg/kg/d). Her blood pressure started to increase following the first methylprednisolone pulse. In addition, two weeks later, severe fluid overload necessitated dialysis. Despite dialysis and the use of four antihypertensive agents (lisinopril, amlodipine, atenolol, doxazosin), her blood pressure fluctuated and remained uncontrolled. A week later, she experienced focal seizures. While a CT brain scan showed no abnormalities, an MRI revealed multifocal areas of bilateral cerebral hemispheric cortico-subcortical signal and diffusion alteration, indicating evolving PRES.

A steroid weaning plan was implemented, and IV cyclophosphamide was initiated according to the National Institutes of Health protocol. Eight weeks later, she had another PRES episode, presenting with seizures confirmed by MRI findings. On both occasions, hypertensive encephalopathy was promptly recognized, and she was transferred to the PICU. Fortunately, her symptoms completely resolved within 12 hours of anticonvulsant and antihypertensive therapies.

**Discussion:**

Common triggers of PRES include autoimmune conditions such as systemic lupus erythematosus (SLE), hypertensive encephalopathy, and drug toxicities (mainly cytotoxic and immunosuppressive drugs), all of which interact in lupus patients. However, it remains unclear how each factor contributes to the development of PRES. Additionally, the exact pathophysiological mechanism behind PRES is still debated. The two most widely accepted hypotheses involve: firstly, increased arterial blood pressure overwhelming cerebral autoregulatory mechanisms, leading to vascular leakage and vasogenic oedema. Secondly, endothelial dysfunction, where endothelial damage caused by circulating toxins enhances the release of vasoactive and immunogenic agents, altering vascular permeability.

Our patient’s development of PRES is multifactorial, with lupus nephritis being a primary cause, aggravated by chronic kidney disease, leading to salt and water retention, which was further exacerbated by prednisolone. Other drugs, such as rituximab and cyclophosphamide, might have also been implicated in causing hypertension.

PRES following rituximab infusion has been documented, with onset ranging from immediately after infusion to up to four weeks later. Proposed mechanisms include compromised blood-brain barrier integrity and immune dysregulation. Intravenous cyclophosphamide has also been identified as a potential trigger for PRES in patients with and without SLE, though the exact reason for this remains unclear.

The dual role of these drugs in potentially precipitating life-threatening risks like PRES while being therapeutic agents presents a confusing dilemma. Much remains to be learned to enable the prevention and prompt response to PRES.

**Key learning points:**

• Juvenile lupus with PRES is an uncommon neurological manifestation influenced by multiple factors.

• Risk factors for PRES in juvenile lupus include lupus nephritis, CKD secondary to lupus, and iatrogenic hypertension including steroids, rituximab, and cyclophosphamide. These factors commonly exist and interplay together.

• The pathogenesis of PRES in lupus, including the role of drugs, remains unclear.

• Patients with lupus should be closely monitored, particularly their blood pressure.

• Early diagnosis and prompt treatment of PRES can reverse radiologic and neurologic findings, leading to a favourable prognosis.

